# Molecular relationships of *Campomanesia xanthocarpa* within Myrtaceae based on the complete plastome sequence and on the plastid *ycf2* gene

**DOI:** 10.1590/1678-4685-GMB-2018-0377

**Published:** 2020-06-10

**Authors:** Lilian de Oliveira Machado, Leila do Nascimento Vieira, Valdir Marcos Stefenon, Helisson Faoro, Fábio de Oliveira Pedrosa, Miguel Pedro Guerra, Rubens Onofre Nodari

**Affiliations:** 1 Universidade Federal de Santa Catarina, Centro de Ciências Agrárias, Departamento de Fitotecnia, Florianópolis, SC, Brazil; 2 Universidade Federal do Paraná, Centro Politécnico, Curitiba, PR, Brazil; 3 Universidade Federal do Pampa, Campus São Gabriel, São Gabriel, RS, Brazil; 4 Fundação Oswaldo Cruz, Instituto Carlos Chagas, Curitiba, PR, Brazil

**Keywords:** Myrteae, guabiroba, plastid, ycf2, evolution

## Abstract

Plastomes are very informative structures for comparative phylogenetic and evolutionary analyses. We sequenced and analyzed the complete plastome of *Campomanesia xanthocarpa* and compared its gene order, structure, and evolutionary characteristics within Myrtaceae. Analyzing 48 species of Myrtaceae, we identified six genes representing ‘hotspots’ of variability within the plastomes (*ycf2, atpA, rpoC2, pcbE*, *ndhH* and *rps16*), and performed phylogenetic analyses based on: (i) the *ycf2* gene, (ii) all the six genes identified as ‘hotspots’ of variability, and (iii) the genes identified as ‘hotspots’ of variability, except the *ycf2* gene. The structure, gene order, and gene content of the *C. xanthocarpa* plastome are similar to other Myrtaceae species. Phylogenetic analyses revealed the *ycf2* gene as a promissing region for barcoding within this family, having also a robust phylogenetic signal. The synonymous and nonsynonymous substitution rates and the *Ka/Ks* ratio revealed low values for the *ycf2* gene among *C. xanthocarpa* and the other 47 analyzed species of Myrtaceae, with moderate purifying selection acting on this gene. The average nucleotide identity (ANI) analysis of the whole plastomes produced phylogenetic trees supporting the monophyly of three Myrtaceae tribes. The findings of this study provide support for planning conservation, breeding, and biotechnological programs for this species.

## Introduction

Plastidial genomes (plastomes) are useful tools to perform comparative analyses associated with phylogenetic and evolutionary studies. The relatively small size, mostly uniparental inheritance, high gene synteny, and elevated copy number in green plant cells are the main characteristics that make plastids useful for such studies. In seed plants, plastome sizes range from 70 to 218 Kbp (Xiao-Ming *et al.*, 2017) and typically present a quadripartite structure with two inverted repeat regions (IRs) divided between the large (LSC) and the small (SSC) single-copy regions (Bock, 2007).

The plastome contains essential genes in conserved open reading frames (ORFs). However, some plastidial ORFs have unknown function and are called hypothetical chloroplast open reading frame (*ycf*). The largest plastome coding sequence (ORF2280 or *ycf2*) encodes a plastidial protein (Glick and Sears, 1993) whose function has been hypothesized to exhibit similarities with *fstH*, such as ATPase-related activities, chaperone function, and activity associated with cell division (Wolfe, 1994).

The elevated substitution rates of the *ycf2* gene led to a pseudogenization process (Downie *et al.*, 1994; Oliver *et al.*, 2010; Wolf *et al.*, 2011). This gene is absent in some plastomes (Downie *et al.*, 1994; Millen *et al.*, 2001), especially in monocot grasses, such as maize, rice, and sugarcane (Maier *et al.*, 1995; Matsuoka *et al.*, 2002; Asano *et al.*, 2004). Thus, the high variability of *ycf2* makes it a potential candidate for species-level DNA barcoding (Kumar *et al.*, 2009).

Investigating the sequence variation and phylogenetic characteristics of the *ycf2* gene in angiosperms, Huang *et al.* (2010) showed that it provides generally well-supported phylogenies, consistent with those inferred from the most comprehensive multigene data. Within angiosperms, Myrtaceae is a large family of shrubs and trees with well-known ecological and economic importance in tropical and subtropical regions of the globe. According to The Plant list (2013), this family is composed by 144 genera and 5,970 species distributed across the world, with predominantly Neotropical and Southern Hemisphere distribution. Its main center of diversity is the wet tropics, predominantly in South America, Australia, and tropical Asia (Grattapaglia *et al.*, 2012). Evolutionary and phylogenetic trends within this family have been studied using single and combined plastidial genes, as well as by complete plastome sequencing (Steane, 2005; Bayly *et al.*, 2013, 2016; Jo *et al.*, 2016; Eguiluz *et al.*, 2017; Machado *et al.*, 2017).

*Campomanesia xanthocarpa* Berg. is a fruit tree species of the family Myrtaceae, native to South America, occurring in Brazil, Argentina, Uruguay, and Paraguay (Lorenzi, 1992). In addition to feeding several mammal and bird species, its fruits are appreciated by local people, being consumed fresh and as component of cakes, ice cream, and beverages. As a heliophyte species, *C. xanthocarpa* is indicated for recovery of degraded areas and as an ornamental plant (Souza and Lorenzi, 2005). Controlled studies have shown that extracts of *C. xanthocarpa* leaves induced reduction in weight gain and glycemia in rats (Biavatti *et al.*, 2004) and were efficient in avoiding gastric ulceration (Markman *et al.*, 2004). In hypercholesterolemic human patients, encapsulated dried leaves of *C. xanthocarpa* significantly decreased total cholesterol and LDL levels (Klafke *et al.*, 2010). This plant also demonstrated anti-inflammatory (Klafke *et al.*, 2016), antimicrobial (Capeletto *et al.*, 2016) and anti-oxidant (Viecili *et al.*, 2014) activities, and may also have therapeutic applications during pregnancy, reducing reabsorption sites, increasing placenta weight and the number of live fetuses (Auharek *et al.*, 2013).

In an effort to elucidate the evolutionary history of South American tree species, the plastome of different native species has been sequenced (Vieira *et al.*, 2014b, 2016a,b; Lopes *et al.*, 2018a,b), including species from the Myrtaceae family (Machado *et al.*, 2017). Aiming to contribute to this attempt and to generate useful information for future efforts towards biotechnology, breeding, and genetic conservation of *C. xanthocarpa,* we used a next-generation sequencing technology to sequence the complete plastome of this species and describe here its genome structure and gene content. Considering previous studies on plastidial genomes, suggesting strong signatures of positive selection between close-related species of tribe Myrteae, this study aimed to answer three main questions: (i) Does the *C. xanthocarpa* plastome resemble the plastidial structure of the species from the tribe Myrteae and family Myrtaceae? (ii) Does the *C. xanthocarpa ycf2* gene present signatures of positive selection within the tribe Myrteae and family Myrtaceae? (iii) Does the *ycf2* gene have a strong taxonomic/phylogenetic signal for resolving relationships within the tribe Myrteae and family Myrtaceae compatible to classic plastidial phylogenies?

## Material and Methods

### Plant material and plastidial DNA isolation

For plastidial DNA isolation, fresh leaves were collected from a single individual of *C. xanthocarpa* in the Department of Botany, Federal University of Santa Catarina (UFSC), Brazil (27º36.094” S, 48º31.310” W). The plastidial DNA was obtained according to Vieira *et al.* (2014a), with the modification that plastidial lysis was achieved by incubating the chloroplast pellet with 8 mL of DNA isolation buffer [1.5 mL 20% SDS, 450 μL 2-mercaptoethanol, and 50 μL proteinase K (10 mg/mL)] in a centrifuge tube at 55 °C for 4 h or overnight.

### Plastome assembly and annotation

The sequencing libraries were prepared using 1 ng of plastidial DNA with the Nextera XT DNA Sample Prep Kit (Illumina Inc., San Diego, CA), according to the manufacturer's instructions. Libraries were sequenced using the MiSeq Reagent Kit v3 (600 cycles) on an Illumina MiSeq Sequencer (Illumina Inc., San Diego, California, USA). The obtained paired-end reads (2 x 300 bp) were used for *de novo* assembly performed with CLC Genomics Workbench v8.0.1. The same software was used to estimate plastome coverage. Initial annotation of the *C. xanthocarpa* plastome was performed using DOGMA - Dual Organellar GenoMe Annotator (Wyman *et al.*, 2004). From this initial annotation, putative start, stop, and intron positions were determined based on comparisons to homologous genes in other plastomes. The tRNA genes were further verified using tRNAscan-SE (Schattner *et al.*, 2005). A circular plastome map was drawn using OGDRAW - OrganellarGenomeDRAW (Lohse *et al.*, 2007). REPuter (Kurtz and Schleiermacher, 1999) was used to identify and locate the IRs in the *C. xanthocarpa* plastome by forward versus reverse complement (palindromic) alignment, with minimal repeat size was set to 30 bp, and identity of repeats ≥ 90%. REPuter was also used to identify and locate LSC/IRb/SSC/IRa sizes and boundaries in 12 other previously published chloroplast genomes.

The complete *C. xanthocarpa* plastome sequence reported in this study was deposited in the GenBank database under accession number KY392760.

### Taxonomical relationships within Myrtaceae based on the whole plastome

Aiming to investigate the taxonomical relationships within Myrtaceae based on the whole plastome sequences, we employed the average nucleotide identity (ANI) analysis. This analysis is a measure of nucleotide-level genomic similarity between two genomes, where the averages reflect the degree of divergence between coding regions of the compared genomes and, consequently, evolutionary distances between these genomes. It consists in calculating the percentage nucleotide identity of the matching regions of two genomes, as an average for all matching regions.

Sequences from plastomes of 47 Myrtaceae species including the *C. xanthocarpa* plastome reported in the present study and 46 Myrtaceae species, which have whole plastome sequences deposited in the GenBank database, were used in this analysis, including 30 species of genus *Eucalyptus*, six of the genus *Corymbia*, two of the genus *Angophora*, and one each of the *Allosyncarpia, Eugenia, Stockwellia, Syzygium*, *Acca, Pimenta, Plinia* and *Psidium* genera (Table 1). The plastome sequence of *Lagerstroemia fauriei* (Myrtales: Lythraceae) was used as outgroup.

**Table 1:** Comparison of chloroplast genomes of Myrtaceae species and outgroup analyzed in this study.

Species	Accession	Size	LSC b	SSC c	IR d
*Campomanesia xanthocarpa* a	KY392760	158,131	87,596	18,595	25,970
*Acca sellowiana*	KX289887	159,370	88,028	18,598	26,372
*Allosyncarpia ternate*	KC180806	159,593	88,218	18,571	26,402
*Angophora costata*	KC180805	160,326	88,769	18,773	26,392
*Angophora floribunda*	KC180804	160,245	88,715	18,746	26,392
*Corymbia eximia*	KC180802	160,012	88,522	18,672	26,409
*Corymbia gummifera*	KC180800	160,713	88,310	17,197	27,603
*Corymbia henryi*	KP015032	160,095	88,589	18,688	26,409
*Corymbia maculate*	KC180801	160,045	88,557	18,670	26,409
*Corymbia tessellaris*	KC180803	160,127	88,617	18,692	26,409
*Corymbia torelliana*	KP015033	159,994	88,494	18,682	26,409
*Eucalyptus aromaphloia*	KC180789	160,149	88,925	18,468	26,378
*Eucalyptus baxteri*	KC180773	160,032	88,926	18,368	26,369
*Eucalyptus camaldulensis*	KC180791	160,164	88,874	18,492	26,399
*Eucalyptus cladocalyx*	KC180786	160,213	89,045	18,376	26,396
*Eucalyptus cloeziana*	KC180779	160,015	88,867	18,446	26,351
*Eucalyptus curtisii*	KC180782	160,038	88,828	18,448	26,381
*Eucalyptus deglupta*	KC180792	160,177	88,936	18,425	26,408
*Eucalyptus delegatensis*	KC180771	159,724	88,490	18,498	26,368
*Eucalyptus diversicolor*	KC180795	160,214	88,994	18,416	26,402
*Eucalyptus diversifolia*	KC180774	159,954	88,901	18,315	26,369
*Eucalyptus elata*	KC180776	159,899	88,762	18,401	26,368
*Eucalyptus erythrocorys*	KC180799	159,742	88,691	18,287	26,382
*Eucalyptus globulus*	AY780259	160,286	89,012	18,488	26,393
*Eucalyptus grandis*	HM347959	160,137	88,872	18,475	26,395
*Eucalyptus guilfoylei*	KC180798	160,520	89,054	18,096	26,685
*Eucalyptus marginata*	KC180781	160,076	88,828	18,476	26,386
*Eucalyptus melliodora*	KC180784	160,386	89,073	18,557	26,378
*Eucalyptus microcorys*	KC180797	160,225	89,051	18,410	26,382
*Eucalyptus nitens*	KC180788	160,271	89,005	18,468	26,399
*Eucalyptus obliqua*	KC180769	159,527	88,293	18,498	26,368
*Eucalyptus patens*	KC180780	160,187	88,902	18,543	26,371
*Eucalyptus polybractea*	KC180785	160,268	88,944	18,530	26,397
*Eucalyptus radiate*	KC180770	159,529	88,295	18,498	26,368
*Eucalyptus regnans*	KC180777	160,031	88,860	18,447	26,362
*Eucalyptus saligna*	KC180790	160,015	89,041	18,426	26,274
*Eucalyptus salmonophloia*	KC180796	160,413	89,173	18,466	26,387
*Eucalyptus sieberi*	KC180775	159,985	88,848	18,401	26,368
*Eucalyptus spathulata*	KC180793	161,071	88,729	17,116	27,613
*Eucalyptus torquata*	KC180794	160,223	89,018	18,439	26,383
*Eucalyptus umbra*	KC180778	159,576	88,864	18,658	26,027
*Eucalyptus verrucata*	KC180772	160,109	88,890	18,481	26,369
*Eugenia uniflora*	KR867678	158,445	87,459	18,318	26,334
*Pimenta dioica*	KY085891	158,984	87,572	18,586	26,413
*Plinia trunciflora*	KU318111	159,512	88,097	18,587	26,414
*Psidium guajava*	KX364403	158,841	87,675	18,464	26,351
*Stockwellia quadrifida*	KC180807	159,561	88,247	18,544	26,385
*Syzygium cumini*	GQ870669	160,373	89,081	18,508	26,392
*Lagerstroemia fauriei* (Lythraceae)^e^	KT358807	152,440	83,923	16,933	25,792

a Species with plastid genomes sequenced in this study

b Large Single Copy Region

c Small Single Copy Region

d Inverted Repeat Region

e Outgroup

ANI was calculated for the whole plastomes using the Pyani script (Python module) for average nucleotide identity analyses; (https://github.com/widdowquinn/pyani), aligning the sequences with the MUMmer algorithm (Goris *et al.*, 2007).

### Evolutionary and phylogenetic patterns within Myrtaceae based on the *ycf2* gene

In order to evaluate the phylogenetic signal of the *ycf2* gene within Myrtaceae, we analyzed evolutionary and phylogenetic patterns of this gene among representatives of this family using sequences obtained from the same species employed in the ANI analysis (Table 1).

The evolutionary patterns of the plastidial *ycf2* gene in family Myrtaceae were evaluated by estimating the *Ka/Ks* ratio for the *ycf2* protein-coding gene. The evolutionary characteristics, nonsynonymous (*Ka*) and synonymous substitution rates (*Ks*), as well as *Ka/Ks* ratio, were calculated using Model Averaging in the KaKs_Calculator program (Zhang *et al.*, 2006). The genes were pairwise aligned using the MUltiple Sequence Comparison by Log-Expectation (MUSCLE) algorithm (Edgar, 2004) to identify synonymous and nonsynonymous substitution.

For the phylogenetic relationships of Myrtaceae based on the *ycf2* gene, sequences were aligned by Multiple Alignment using Fast Fourier Transform -MAFFT (Katoh and Standley, 2013). The substitution model was selected by means of the Akaike information criterion using jModelTest (Darriba *et al.*, 2012) with seven substitution schemes, as this set covers all the possible models present in MrBayes software. Bayesian inference was conducted using MrBayes v3.2.6 at CIPRES Science Gateway V. 3.3, with the general time reversible (GTR) model of substitution incorporating invariant sites (GTR + I), as suggested by the model test selection. Markov Chain Monte Carlo (MCMC) simulations were run for 6,000,000 generations (average standard deviation of split frequencies = 0.003027), discarding the first 25% of trees as burn-in. The remaining trees were represented and edited using FigTree v1.4.1.

In addition, we performed a sliding window analysis of the total plastid genome of all analyzed species using the software DNAsp v.5 (Librado and Rozas, 2009). The window length and the step size were set as 200 and 50 bp, respectively. The genes representing hotspots of sequence divergence were identified and used for a new phylogenetic analysis, both including and excluding the *ycf2* region, in order to evaluate the individual contribution of this gene to the phylogenetic patterns in comparison to other plastid genes highly variable within Myrtaceae.

## Results

### Sequencing results

The Illumina MiSeq sequencing resulted in a high plastome coverage (∼370x) with a total of 3,240,548 raw reads, an average read length of 147.9 bp, and a total number of 479,391,832 base pairs. After trimming (quality score limit of 0.05) a total of 3,228,689 reads were mapped in aligned pairs with mean length of 147.52 bp, generating a total of 58,815,423 bp, which were used for the *de novo* assembly.

### General features of the *Campomanesia xanthocarpa* plastome

**Figure 1:**
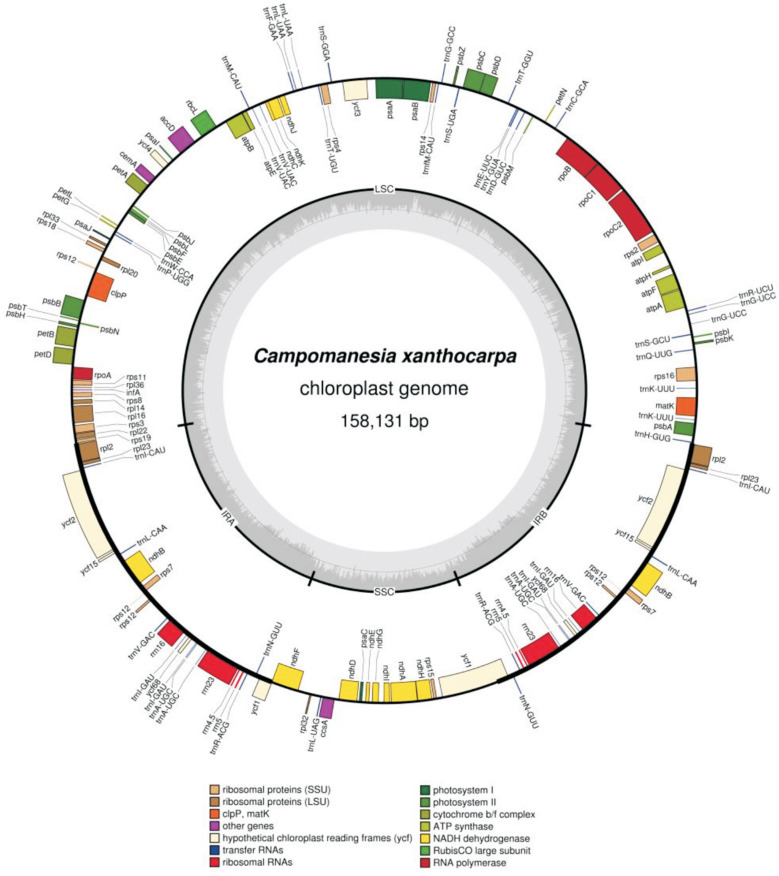
Gene map of the *Campomanesia xanthocarpa* chloroplast genome. Genes drawn inside the circle are transcribed clockwise, and genes drawn outside are transcribed counterclockwise. Genes belonging to different functional groups are color-coded. The darker gray in the inner circle corresponds to GC content, and the lighter gray corresponds to AT content.

The *C. xanthocarpa* plastome has 158,131 bp in length, with a GC content of 36.98% and the general quadripartite structure, consisting of a pair of IRs (25,970 bp) separated by the LSC (87,596 bp) and SSC (18,595 bp) regions (Figure 1; Table 2). It is the smallest plastidial genome size within the reported plastomes of Myrtaceae, 2,582 bp shorter than the plastome of *Corymbia gummifera*, the longest plastidial genome reported for this family. The IR region of the *C. xanthocarpa* plastome is the shortest among the 48 Myrtaceae species evaluated herein (Table 2).

**Table 2:** Summary of *Campomanesia xanthocarpa* chloroplast genome characteristics.

Characteristics of plastome	*C. xanthocarpa*
Plastome Size (bp)	158,131
LSC size in bp (%)	87,596 (55.39)
SSC size in bp (%)	18,595 (11.76)
IR length in bp	25,970
Different genes	115
Different PCG	77
Different tRNA genes	30
Different rRNA genes	4
Different pseudogenes	4
Different genes duplicated by IR	20
Different genes with introns	18
Overall % GC content	36.98
% GC content in LSC	34.8
% GC content in SSC	30.6
% GC content in IR	42.9

PCG = Protein-coding gene

The plastome contains 112 genes and four pseudogenes, with the same gene order and gene clusters as other Myrtaceae. The presence of pseudogenes is a known feature of Myrtaceae plastomes. Out of the 112 genes, 90 were single copy and 19 were duplicated (Figure 1; Table S1). In addition, 18 were intron-containing genes (Table 3), including nine protein-coding genes with a single intron, two protein-coding genes with a double intron, six tRNA genes with a single intron and one trans-splicing gene (*rps12*). Among intron-containing genes, 12 are located in the LSC region, one in the SSC region, and four in the IR region.

**Table 3:** Genes with introns in *Campomanesia xanthocarpa* chloroplast genome and length of exons and introns.

Region	Gene	Exon I (bp)	Intron I (bp)	Exon II (bp)	Intron II (bp)	Exon III (bp)
LSC	*rps16*	206	889	38		
LSC	*rpoC1*	1616	730	452		
LSC	*atpF*	410	747	146		
LSC	*petB*	5	772	647		
LSC	*petD*	8	753	473		
LSC	*rpl16*	398	1005	8		
LSC	*ycf3*	152	725	227	760	125
LSC	*clpP*	227	620	290	871	68
LSC	*trnK-UUU*	34	2530	36		
LSC	*trnG-UCC*	22	750	48		
LSC	*trnV-UAC*	36	595	38		
LSC	*trnL-UAA*	36	504	49		
SSC	*ndhA*	539	1061	551		
LSC/IRs	*rps12* *	113	–	209	–	26
IR	*rpl2*	434	662	392		
IR	*ndhB*	755	695	776		
IR	*trnI-GAU*	36	956	33		
IR	*trnA-UGC*	37	804	34		

* rps12 is trans-spliced with the 5’-end located in the LSC region and the duplicated 3’-end in the IR regions

The LSC/IRb/SSC/IRa boundary regions were examined to compare four junctions (JLA, JLB, JSA, and JSB) among 12 species of three tribes of Myrtaceae and an outgroup, from Lythraceae family. The IR lengths ranged from 25,792 bp to 26,414 bp, and the position of IRs boundaries varied for each species. The boundary between LSC and IR regions was located within the *rps19* gene, resulting in the formation of an *rps19* pseudogene in *C. xanthocarpa*, *A. ternata*, *S. quadrifida*, *P. dioica*, *P. trunciflora*, *P. guajava* and *L. fauriei* chloroplast genomes (Figure S1). In the other six species, the LSC comprises an intact *rps19* gene together with 2 bp (*E. grandis*), 3 bp (*A. sellowiana* and *E. uniflora*), 6 bp (*S. cumini*), and 8 bp (*A. costata* and *C. maculata*) of non-coding region beyond the LSC-IRb border. The IRa-LSC border in these six species is located in the intergenic spacer (IGS) between *rpl2* and *trnH*. The *trnH* gene in *C. xanthocarpa, A. ternata, S. quadrifida, P. dioica*, *P. trunciflora, P. guajava* and *L. fauriei* extends to the IRa by 31 bp, 5 bp, 5 bp, 1 bp, 4 bp, 11 bp and 3 bp, respectively, whereas the same gene for *A. sellowiana*, *E. uniflora*, *A. costata* and *C. maculata*, *E. grandis*, and *S. cumini* is, respectively 53 bp, 44 bp, 9 bp, 9 bp, 2 bp, and 56 bp away from the IRa-LSC border. The boundary of the SSC-IRb junction in Myrtales plastomes was located within the *ycf1* gene, also resulting in the formation of a *ψ*ycf1 pseudogene, which varied in length between 1,007 bp and 2,251 bp. In the *A. ternata* and *L. fauriei* chloroplast genomes, the *ndhF* gene at the IRb-SSC border extends 35 bp and 38 bp into the IRb region, respectively. This gene is located in the SSC region in the 11 other species, and is separated from the IRb-SSC border by five to 225 bp.

### Taxonomic relationships within Myrtaceae based on the whole plastome

**Figure 2:**
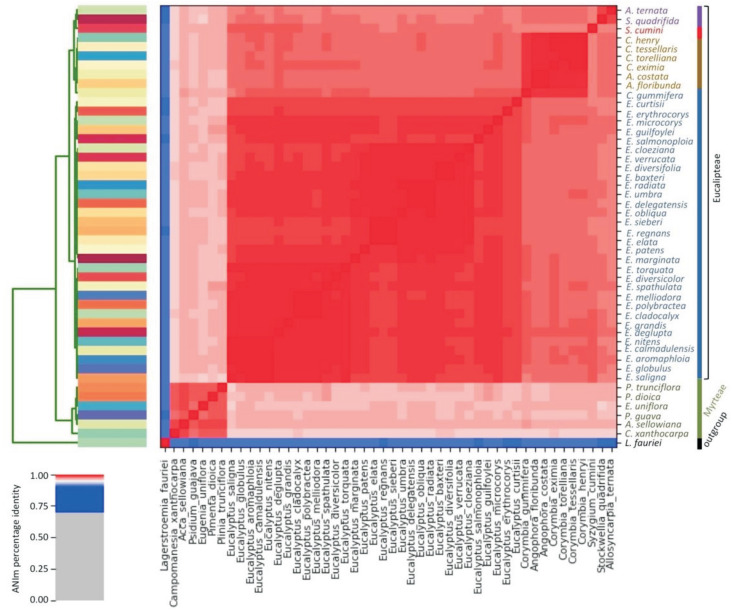
Heatmap of ANIm percentage identity for 48 Myrtaceae species and the outgroup species *Lagerstroemia fauriei* (Myrtales: Lythraceae; KT358807). Cells in the heatmap corresponding to 95% ANIm sequence identity are in red color; Cells corresponding to 75% ANIm sequence identity are in blue. Color intensity fades as the comparisons approach 95% ANIm sequence identity. Color bars above and to the left of the heatmap correspond to the species level that was analyzed.

The average nucleotide identity (ANI) analysis represents a mean of identity values between homologous regions shared by two genomes, and was used to compare the complete plastomes of 47 species of Myrtaceae and *Lagerstroemia fauriei* as outgroup species. The plastomes sequence analysis (∼160 kb) indicated an ANI above the 95% threshold for all species within Myrtaceae. Despite the high overall ANI value observed, two major clades can be identified in the dendrogram and are closely apportioned in the heatmap (Figure 2). The Myrteae clade consists of six species [*Campomanesia* + [[*Acca* + *Psidium*] + [[*Pimenta* + *Plinia*] + *Eugenia*]]]. The second clade, which includes species from both tribes Syzygieae and Eucalypteae, is subdivided in four subgroups, one containing all *Eucalyptus* species (all pairwise comparisons with ANI values near 100%), a second with representatives of *Stockwellia* and *Allosyncarpia,* a third subgroup comprising *Corymbia* and *Angophora,* and a fourth subgroup with the unique *Sysygium* species, from tribe Syzygieae (Figure 2).

### Evolutionary and phylogenetic patterns within Myrtaceae based on the *ycf2* gene

**Figure 3:**
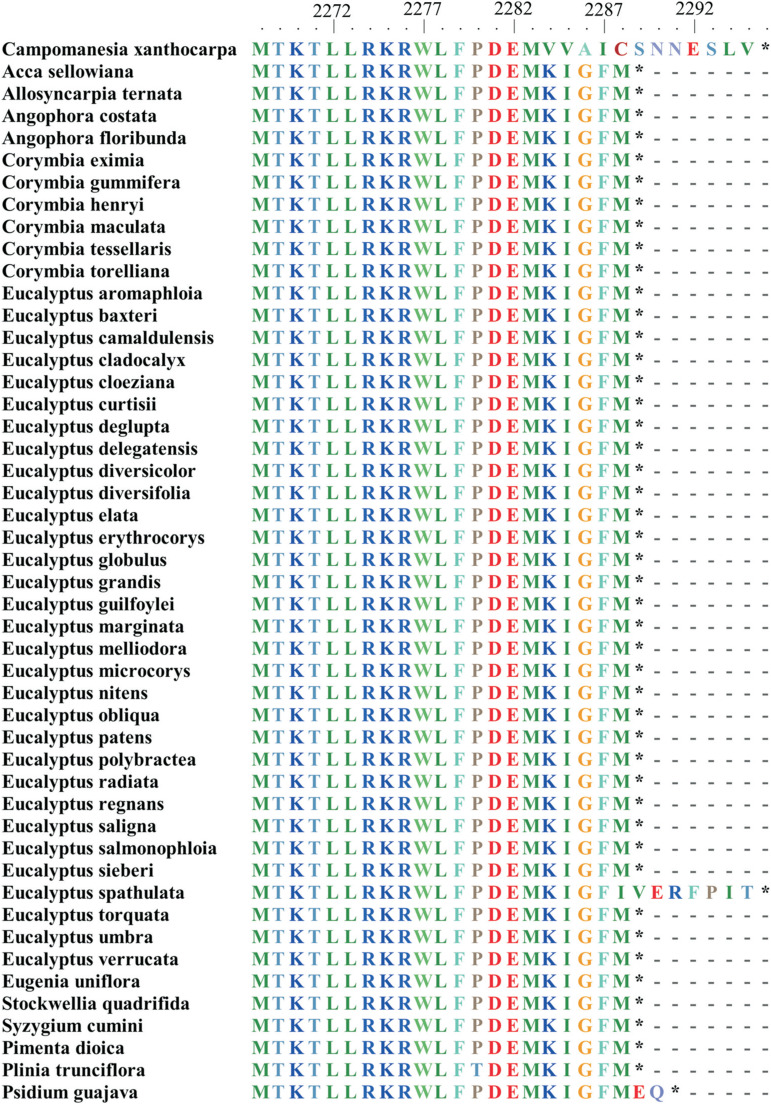
Comparison of the C-terminal region of the amino acid sequences of the *ycf2* gene in 47 species of Myrtaceae.

The translated product of *ycf2* from the plastome of *C. xanthocarpa* contains 2,295 amino acids (6,885 bp). It is longer than the sequence reported from all other species of Myrtaceae included in this study (ranging from 2,288 to 2,289 amino acids), except for *Eucalyptus spathulata*. This difference is observed in the C-terminal portion of the translated product (Figure 3). The translated product of this gene in *C. xanthocarpa* has the same amino acid sequence as the other species up to position 2,283 (Figure 3). The amino acids from position 2,284 to 2,288 are conserved in all other species, with exception of *E. spathulata*. The amino acids of positions 2,289 and 2,290 in *P. guajava* and from positions 2,289 to 2,295 in *E. spathulata* are different from the amino acids of *C. xanthocarpa* (Figure 3). The analysis of the pairwise diversity between *C. xanthocarpa* and 46 species of Myrtaceae (Table 4) showed a *Ka* ranging from 0.0019 (*Pimenta dioica*) to 0.0054 (*Eucalyptus grandis, E. deglupta* and *Corymbia gummifera*) with a mean *Ka* = 0.0048. Estimations of *Ks* ranged from 0.0010 (*Acca sellowiana* and *Plinia trunciflora*) to 0.0138 (*Eucalyptus erythrocorys*), with a mean *Ks* = 0.0114. The average *Ka/Ks* ratio was 0.4620, ranging from 0.03207 (*Stockwellia quadrifida*) to 3.0 (*Plinia trunciflora*).

**Table 4:** Comparison of polymorphisms (segregating sites), nonsynonymous (*Ka*) and synonymous (*Ks*) substitution rates and *Ka/Ks* ratio of the *ycf2* gene among *Campomanesia xanthocarpa* and 46 species of Myrtaceae.

Species	*C. xanthocarpa*/Species			
	Polymorphisms	*Ka*	*Ks*	*Ka/Ks*
Tribe Myrteae				
*Acca sellowiana*	13	0.0021	0.0010	2.0114
*Eugenia uniflora*	15	0.0021	0.0028	0.7413
*Pimenta dioica*	13	0.0019	0.0020	0.9677
*Plinia trunciflora*	19	0.0032	0.0010	3.0000
*Psidium guajava*	26	0.0035	0.0049	0.7127
Tribe Eucalypteae				
*Corymbia eximia*	40	0.0046	0.0118	0.3921
*Corymbia gummifera*	42	0.0054	0.0101	0.5314
*Corymbia henryi*	42	0.0051	0.0110	0.4677
*Corymbia maculata*	42	0.0051	0.0110	0.4677
*Corymbia tessellaris*	43	0.0052	0.0117	0.4420
*Corymbia torelliana*	44	0.0052	0.0127	0.4053
*Eucalyptus aromaphloia*	42	0.0050	0.0116	0.4309
*Eucalyptus baxteri*	40	0.0046	0.0118	0.3921
*Eucalyptus camaldulensis*	43	0.0052	0.0117	0.4433
*Eucalyptus cladocalyx*	44	0.0052	0.0127	0.4062
*Eucalyptus cloeziana*	40	0.0046	0.0118	0.3921
*Eucalyptus curtisii*	44	0.0053	0.0121	0.4403
*Eucalyptus deglupta*	44	0.0054	0.0115	0.4704
*Eucalyptus delegatensis*	41	0.0048	0.0119	0.4043
*Eucalyptus diversicolor*	44	0.0052	0.0124	0.4199
*Eucalyptus diversifolia*	41	0.0048	0.0116	0.4189
*Eucalyptus elata*	40	0.0046	0.0118	0.3921
*Eucalyptus erythrocorys*	46	0.0053	0.0138	0.3811
*Eucalyptus globulus*	43	0.0052	0.0114	0.4584
*Eucalyptus grandis*	45	0.0054	0.0125	0.4318
*Eucalyptus guilfoylei*	41	0.0048	0.0119	0.4038
*Eucalyptus marginata*	42	0.0050	0.0117	0.4302
*Eucalyptus melliodora*	45	0.0053	0.0128	0.4173
*Eucalyptus microcorys*	39	0.0044	0.0121	0.3655
*Eucalyptus nitens*	42	0.0050	0.0116	0.4309
*Eucalyptus obliqua*	41	0.0048	0.0119	0.4043
*Eucalyptus patens*	39	0.0046	0.0108	0.4304
*Eucalyptus polybractea*	44	0.0052	0.0127	0.4062
*Eucalyptus radiata*	41	0.0048	0.0119	0.4043
*Eucalyptus regnans*	41	0.0046	0.0128	0.3594
*Eucalyptus saligna*	42	0.0051	0.0119	0.4283
*Eucalyptus salmonophloia*	44	0.0050	0.0136	0.3641
*Eucalyptus sieberi*	41	0.0046	0.0128	0.3591
*Eucalyptus spathulata*	44	0.0053	0.0121	0.4362
*Eucalyptus torquata*	43	0.0052	0.0117	0.4429
*Eucalyptus umbra*	42	0.0050	0.0120	0.4162
*Eucalyptus verrucata*	41	0.0048	0.0116	0.4186
*Stockwellia quadrifida*	34	0.0037	0.0115	0.3207
Tribe Syzygieae				
*Syzygium cumini*	35	0.0043	0.0093	0.4587

**Figure 4:**
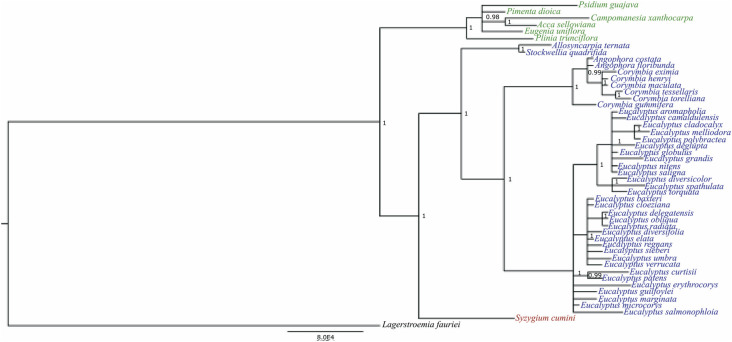
Bayesian phylogeny based on the cp ycf2 sequence of 48 Myrtaceae species and the outgroup species *Lagerstroemia fauriei* (Myrtales: Lythraceae; KT358807). Branch length is proportional to the inferred divergence level. The scale bar indicates the number of inferred nucleic acid substitutions per site.

The matrix of the *ycf2* gene used for the phylogenetic inference was composed of 6,969 nucleotide positions, and the Bayesian phylogenetic tree with highest log-likelihood (lnL = −11,575.58) is shown in Figure 4. Monophyly of the tribes of Myrteae and Eucalypteae was confirmed with posterior probability (PP) of 1.0 (Figure 4). The tribe Syzygieae is sister to Eucalypteae with PP = 1.0.

The clades within the Myrteae tribe were highly supported, with posterior probability of 0.9788 for the clade *Eugenia uniflora, Acca sellowiana, Campomanesia xanthocarpa, Pimenta dioica, Plinia trunciflora,* and *Psidium guajava,* and PP = 1.0 for the node that ties *A. sellowiana* and *C. xanthocarpa*. Similarly, the Eucalypteae tribe segregated into two well-supported monophyletic clades: [*Eucalyptus* [*Angophora* + *Corymbia*]] (PP = 1.0) and [*Allosyncarpia ternata* + *Stockwelia quadrifida*] (PP = 1.0).

**Figure 5:**
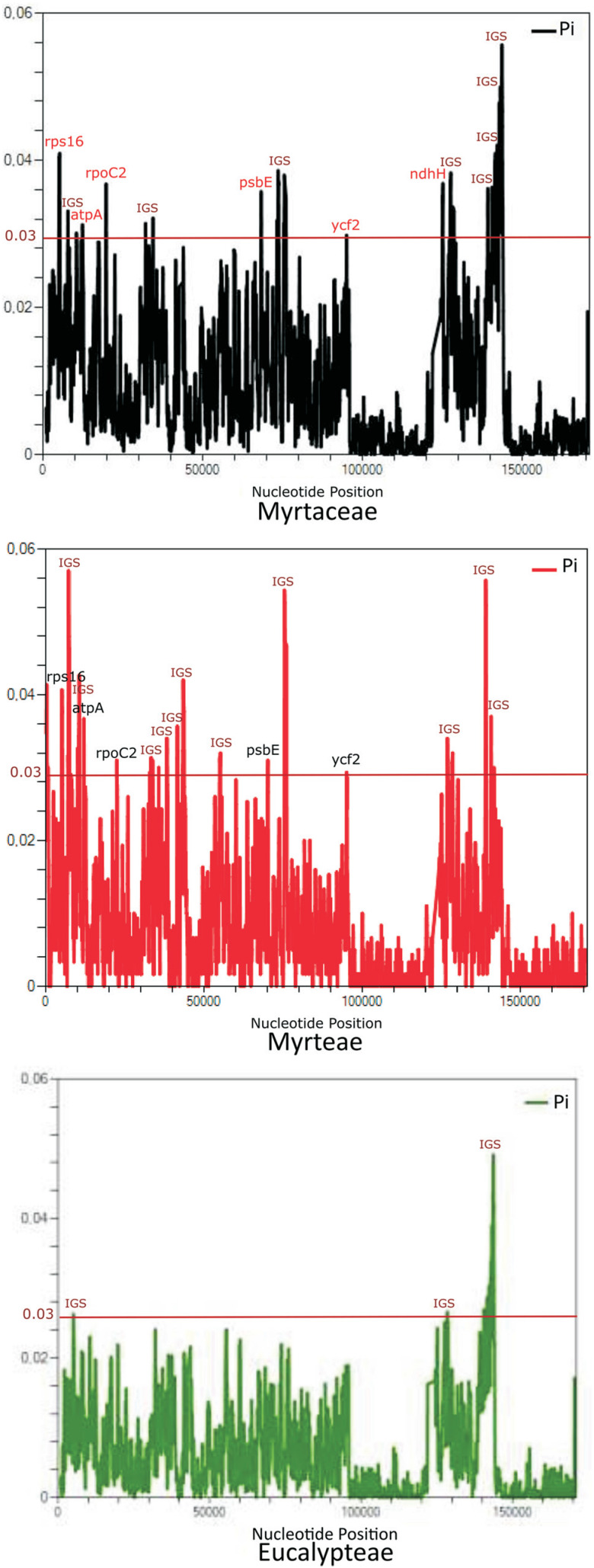
Sliding window analyses of aligned whole plastomes for the family Myrtaceae, the tribe Myrteae and the tribe Eucalypteae. The regions with high nucleotide variability (Pi > 0.03) are indicated. Pi = nucleotide diversity within each window.

The sliding window analysis (Figure 5) revealed six genes as hotspots of sequence divergence (π 3 0.03): *ycf2, atpA, rpoC2, pcbE*, *ndhH,* and *rps16.* The phylogenetic tree constructed with all six genes (Figure S2) revealed the same topology obtained in the tree built only with the *ycf2* gene sequence (Figure 4). When the phylogenetic analysis was performed using the *atpA, rpoC2, pcbE, ndhH,* and *rps16* genes (Figure S3), i.e., excluding the *ycf2* gene sequence, Syzygieae was positioned basal to Myrteae and Eucalypteae, differing from the trees built using all six genes (Figure S2) and based only on the *ycf2* sequence (Figure 4). However, the support for the node of Myrteae in this tree was low (PP = 0.55; Figure S3).

## Discussion

### Structural patterns of the *C. xanthocarpa* plastome in comparison to other Myrtaceae

Conservation of plastome structure has been reported for species of Myrtaceae concerning the total length, as well as the length of the SSC, LSC and IR regions, and the number and position of genes (Machado *et al.*, 2017, Xiao-Ming *et al.*, 2017). Fitting with these previous findings, the assembly and annotation of the *C. xanthocarpa* plastome revealed a general structure, including number and category of the genes, similar to that of other 47 species of Myrtaceae, suggesting overall stability. The reduction of the *C. xanthocarpa* plastome is not related to a loss of genes or pseudogenes. Large expansions and reductions in plastome sizes in higher plants have been attributed to the extension of inverted repeats into neighboring single copy regions (Ravi *et al.*, 2008). The difference between the *C. xanthocarpa* plastome and the plastomes of the other species included in this study, which ranged from 314 bp (in comparison to *E. uniflora*) to 2,940 bp (in comparison to *E. spathulata*), was due to indels occurring in intergenic regions of both, IR and LSC regions of all species, except for *P. dioica* (Table 1).

While the structure of the *C. xanthocarpa* plastome presented such stability, the analysis of the *ycf2* gene suggested the occurrence of different patterns of selection at family and at tribe levels. In this study, we found very low variability in this gene among published plastomes of Myrtaceae species, with 45 species presenting the same length and same amino acid sequences for the *ycf2* gene. However, the *ycf2* gene of *C. xanthocarpa* is 18 bp longer than those of these species, 15 bp longer than that of *Psidium guajava* and has the same size as that of *Eucalyptus spathulata.* Moreover, having the same amino acid sequences does not mean having the same nucleotide sequences, what enables the exploitation of the nucleotide sequences of the *ycf2* gene as a barcode region in plants (Kumar *et al.*, 2009). The *ycf2* gene has an essential, but yet little known function in higher plants. Kikuchi *et al.* (2018) demonstrated the function of a protein encoded by this gene, associated to five related nuclear-encoded FtsH-like proteins, in the translocation of preproteins across the inner membrane of the chloroplast. Silencing or reduction in the mRNA synthesis of this gene has been shown to induce cell apoptosis (Drescher *et al.*, 2000). It seems, however, that the elongation of this gene and the substitution of some amino acids in the C-terminal portion of the sequence has no deleterious effect in *C. xanthocarpa, P. guajava* and *E. spathulata.*

### Modest signatures of positive selection for the *C. xanthocarpa ycf2* gene within Myrtaceae

Increased substitution rates and elevated *Ka/Ks* ratios for similar sets of plastid genes have been reported for several plant species. However, it remains uncertain whether these patterns reflect positive selection, relaxed purifying selection, changes in underlying mutation rates, a breakdown in DNA repair mechanisms, such as gene conversion, or some combination of these (Barnard-Kubow *et al.*, 2014).

Concerning the evolutionary patterns of the *ycf2* gene of *C. xanthocarpa* within Myrtaceae, the results of the present study showed evidence of purifying selection acting over this region. The number of segregating sites, not surprisingly, revealed the higher polymorphism when comparing *C. xanthocarpa* to species of tribe Eucalypteae and Syzygieae. For the species of tribe Myrteae, the highest polymorphism was observed between *C. xanthocarpa* and *P. guajava*, supporting the phylogenetic relationship recovered by the *ycf2* gene alone, and also using the six plastid genes with high diversity (Figure 4).

In relation to tribe Eucalypteae and Syzygieae (*Ka/Ks* ratios < 0.5314), the pattern of purifying selection is more evident, towards equilibrium in relation to other species from the Myrteae tribe (*Ka/Ks* ratio > 0.7127). Machado *et al.* (2017) observed a *Ka/Ks* ratio of 0.30 for this gene by comparing the plastomes of the close-related Myrtaceae species *A. sellowiana* and *E. uniflora*, evidencing purifying selection. Evidence of relaxed purifying selection over the *ycf2* gene was also reported for *Campanulastrum americanum* (Campanulaceae) by Barnard-Kubow *et al.* (2014). However, the estimated *Ka/Ks* ratio is a mean over the full length of the gene and, considering its very large size, some regions of *ycf2* are likely experiencing stronger selection, while other regions are more conserved. This hypothesis has to be closely tested, but the sliding window analysis of Tajima's *D* estimations (data not shown) revealed such a pattern of gene regions with significant (*p* < 0.05) negative values within *ycf2*, suggesting signatures of positive selection.

### Taxonomic and phylogenetic patterns of Myrtaceae as revealed by whole plastome and the *ycf2* gene

The taxonomic patterns within Myrtaceae revealed by the analysis of the whole plastome sequences clearly clustered species at the tribe level, with ANI > 95%. Studies involving prokaryotic organisms with ANI values > 95% indicate that they belong to the same species (Goris *et al.*, 2007; Richter and Rossello-Mora, 2009). In our study, we applied the ANI to investigate the identity level of the whole plastome among Myrtaceae species. This is the first time such an analysis was performed for organellar genomes, and it seems to be a useful approach, since the obtained outcomes fit the results obtained using classical phylogenetic analyses based on plastidial genes.

Similarly, the Bayesian phylogenetic inference using the plastidial *ycf2* gene confirmed the monophyly of the tribes Myrteae, Eucalypteae and Sysygieae, as already suggested through phylogenetic inferences based on plastidial and nuclear genes (Sytsma *et al.*, 2004; Wilson *et al.*, 2005; Biffin *et al.*, 2010; Thornhill *et al.*, 2012, 2015), as well as through combined analysis of plastidial DNA regions (*matK* and *ndhF*) (Biffin *et al.*, 2010), 78 protein-coding and four rRNA genes (Jo *et al.*, 2016), 57 plastidial protein-coding genes (Huang *et al.*, 2013; Eguiluz *et al.*, 2017), and complete plastidial sequences (Machado *et al.*, 2017). Also, the topology of the tribe Eucalypteae is equivalent to that proposed based on plastidial genomes (Bayly *et al.*, 2013, 2016) and on nuclear ribosomal ITS sequences (Parra-O *et al.*, 2006). The *ycf2* gene was one of the six genes with higher polymorphism at family and at tribe levels (Figure 5), corroborating the potential of this plastid region for species-level DNA barcoding, as proposed by Kumar *et al.* (2009).

The internal topology of the tribe Myrteae phylogeny obtained with the *ycf2* gene needs to be evaluated with caution, because it is the most species-rich tribe within Myrtaceae (Thornhill *et al.*, 2015), and we have the *ycf2* sequence from just six species representing this taxon. At a higher level, the phylogeny presented by Lucas *et al.* (2007), based on ITS, ETS, *psbA-trnH* and *matK* sequences places the *Pimenta* group as sister of the *Eugenia* group, while the *Plinia* group is placed externally to these two groups. In the *ycf2* phylogeny, the species from *Pimenta* (*Acca sellowiana, Campomanesia xanthocarpa, Psidium guajava* and *Pimenta dioica*) and *Eugenia* (*Eugenia uniflora*) groups were not separated, and *Plinia trunciflora* was placed externally, as in the phylogeny presented by Lucas *et al.* (2007).

### The shortest plastome and longest gene within Myrtaceae

Addressing three main questions in this study concerning the structure and evolution of the plastome of *C. xanthocarpa*, we highlighted some relevant features. First, although the plastome of *C. xanthocarpa* conserves the same general structure as in other 47 studied species, regarding number and position of genes, it is the shortest recorded plastidial genome within the family.

Second, the *ycf2* gene of *C. xanthocarpa* is the longest among the Myrtaceae species that had sequences of this gene deposited in GenBank at the time of this study. Signatures of moderate purifying selection were observed for the *ycf2* gene of *C. xanthocarpa* within Myrtaceae, more apparent in relation to tribe Eucalypteae and tending to equilibrium relative to tribe Myrteae. In addition, the *ycf2* gene revealed a robust phylogenetic signal at the family level, generating a Bayesian inference of phylogenetic relationships equivalent to the taxonomic classification presented using the whole plastome sequences and average nucleotide identity analysis.

Although in the starting steps, these findings have important implications for thinking about the genetics, evolution, conservation, breeding, and biotechnology of *C. xanthocarpa*, a fruit tree species with high biotechnological and agricultural potential that is still underexploited. Understanding the evolutionary and taxonomic/phylogenetic relationships of this species relative to other species from Myrtaceae enables the elaboration of conservation, breeding and biotechnology programs with a consistent scientific basis. Thus, enterprises towards safeguarding and managing the species’ genetic resources, as well as selecting and developing cultivars for agricultural or biotechnological uses will be greatly benefited by the results of this study. For instance, plastid SSR markers for *C. xanthocarpa* will be soon released by our group, with direct applicability for marker assisted selection.

## Supplementary material

The following supplementary material is available for this article:

Figure S1 Comparison of boundary positions between single copy and inverted repeat regions among 13 chloroplast genomes of Myrtales.

Figure S2 Bayesian phylogeny based on six most variable genes on chloroplast sequences of 48 Myrtaceae species and the outgroup *Lagerstroemia fauriei* (Myrtales: Lythraceae; KT358807).

Figure S3 Bayesian phylogeny based on five most variable genes on chloroplast sequences of 48 Myrtaceae species and the outgroup *Lagerstroemia fauriei* (Myrtales: Lythraceae; KT358807).

Table S1 List of genes identified in *Campomanesia xanthocarpa* chloroplast genome.
