# Supplementary material for: Molecular relationships of Campomanesia xanthocarpa within Myrtaceae based on the complete plastome sequence and on the plastid ycf2 gene
Source: Genet Mol Biol. 2020 Jun 10;43(2):e20180377. doi: 10.1590/1678-4685-GMB-2018-0377 (PMC7288672; doi:10.1590/1678-4685-GMB-2018-0377)
Supplement: Supplementary file 1 [file 1415-4757-GMB-43-2-e20180377-suppl4.pdf]

Supplementary Material to " Molecular relationships of *Campomanesia xanthocarpa* within Myrtaceae based on the complete plastome sequence and on the plastid *ycf2* gene"

Table S1 - List of genes identified in *Campomanesia xanthocarpa* chloroplast genome

**Category of genes**	**Group of genes**	**Name of genes**								
**Self replication**	Large subunit of ribosome	*rpl14*	*rpl16*	*rpl2* [a,b]	*rpl20*	*rpl22*	*rpl23*[b]	*rpl32*	*rpl33*	*rpl36*
	DNA-dependent RNA polymerase	*rpoA*	*rpoB*	*rpoC1*[a]	*rpoC2*					
	Small subunit of ribosome	*rps11*	*rps12*[b,*]	*rps14*	*rps15*	*rps16*[a]	*rps18*	*rps19*	*rps2*	*rps3*
		rps4	*rps7*[b]	*rps8*						
	Ribosomal RNA Genes	*rrn16*[b]	*rrn23*[b]	*rrn4.5*[b]	*rrn5*[b]					
	Transfer RNA Genes	*trnA-UGC* [a,b]	*trnC-GCA*	*trnD-GUC*	*trnE-UUC*	*trnF-GAA*	*trnfM-CAU*	*trnG-GCC*	*trnG-UCC*[a]	*trnH-GUG*
		trnI-CAU [b]	*trnI-GAU* [a,b]	*trnK-UUU*[a]	*trnL-CAA*[b]	*trnL-UAA*[a]	*trnL-UAG*	*trnM-CAU*	*trnN-GUU*[b]	*trnP-UGG*
		trnQ-UUG	*trnR-ACG*[b]	*trnR-UCU*	*trnS-GCU*	*trnS-GGA*	*trnS-UGA*	*trnT-GGU*	*trnT-UGU*	*trnV-GAC*[b]
		trnV-UAC[a]	*trnW-CCA*	*trnY-GUA*						
	Translational initiation factor	*infA* [c]								
**Genes for hotosynthesis**	Subunits of photosystem I	*psaA*	*psaB*	*psaC*	*psaI*	*psaJ*	*ycf3*[a]	*ycf4*		
	Subunits of photosystem II	*psbA*	*psbB*	*psbC*	*psbD*	*psbE*	*psbF*	*psbH*	*psbI*	*psbJ*
		psbK	*psbL*	*psbM*	*psbN*	*psbT*	*psbZ*			
	Subunits of cytochrome	*petA*	*petB*[a]	*petD*[a]	*petG*	*petL*	*petN*			
	Subunits of ATP synthase	*atpA*	*atpB*	*atpE*	*atpF*[a]	*atpH*	*atpI*			
	Large subunit of rubisco	*rbcL*								
	Subunits of NADH-dehydrogenase	*ndhA*[a]	*ndhB*[a,b]	*ndhC*	*ndhD*	*ndhE*	*ndhF*	*ndhG*	*ndhH*	*ndhI*
		ndhJ	*ndhK*							
**Other genes**	Subunit of Acetyl-CoA-carboxylase	*accD*								

Category of genes	Group of genes	Name of genes		
	c-type cytochrome synthesis gene	*ccsA*		
	Envelope membrane protein	*cemA*		
	Protease	*clpP*[a]		
	Maturase	*matK*		
	Component of TIC complex	*ycf1*[b,c*]		
**Genes unknown function**	Unknown function	*ycf2*[b]	*ycf15*[b,c]	*ycf68*[b,c]

[a] Intron-containing gene; [b] Duplicated genes; [c] Pseudogene; [c*] *ycf1* is pseudogene at the boundary between IRB and SSC regions; [*]*rps12* is trans-spliced with the 5′-end located in the LSC region and the duplicated 3′-end in the IR region.
