# Supplementary material for: Molecular relationships of Campomanesia xanthocarpa within Myrtaceae based on the complete plastome sequence and on the plastid ycf2 gene
Source: Genet Mol Biol. 2020 Jun 10;43(2):e20180377. doi: 10.1590/1678-4685-GMB-2018-0377 (PMC7288672; doi:10.1590/1678-4685-GMB-2018-0377)
Supplement: Supplementary file 2 [file 1415-4757-GMB-43-2-e20180377-suppl1.pdf]

Supplementary Material to "Molecular relationships of *Campomanesia xanthocarpa* within Myrtaceae based on the complete plastome sequence and on the plastid *ycf2* gene"

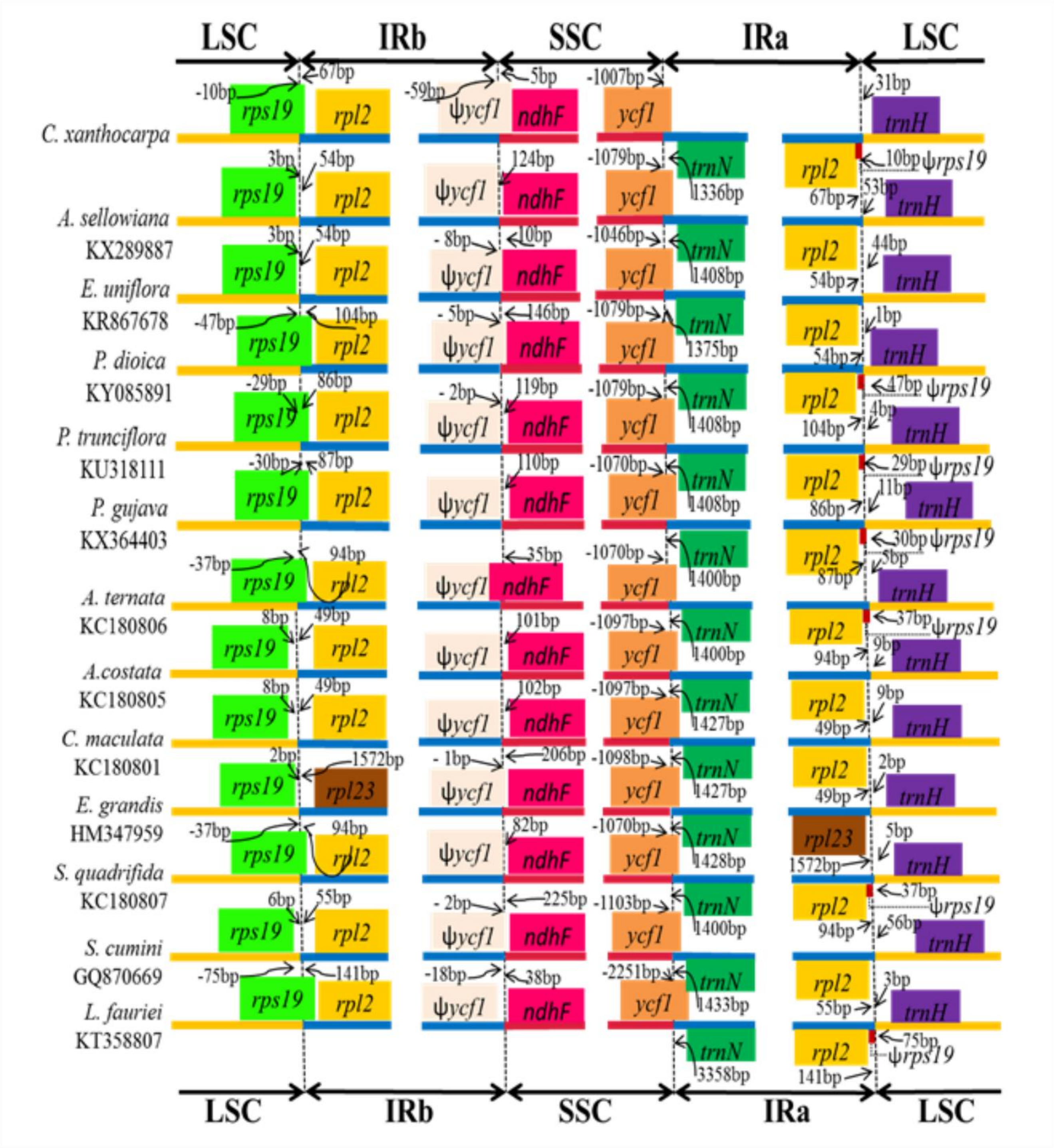

Figure S1 - Comparison of boundary positions between single copy (large, LSC, or small, SSC) and inverted repeat (IR) regions among 13 chloroplast genomes of Myrtales. Genes above lines are transcribed clockwise, while genes below the lines are transcribed counterclockwise. Ψ indicates a pseudogene
