# Supplementary material for: Molecular relationships of Campomanesia xanthocarpa within Myrtaceae based on the complete plastome sequence and on the plastid ycf2 gene
Source: Genet Mol Biol. 2020 Jun 10;43(2):e20180377. doi: 10.1590/1678-4685-GMB-2018-0377 (PMC7288672; doi:10.1590/1678-4685-GMB-2018-0377)
Supplement: Supplementary file 3 [file 1415-4757-GMB-43-2-e20180377-suppl2.pdf]

*Genetics and Molecular Biology* 43, 2 (2020)

# Supplementary Material to "Molecular relationships of *Campomanesia xanthocarpa* within Myrtaceae based on the complete plastome sequence and on the plastid *ycf2* gene"

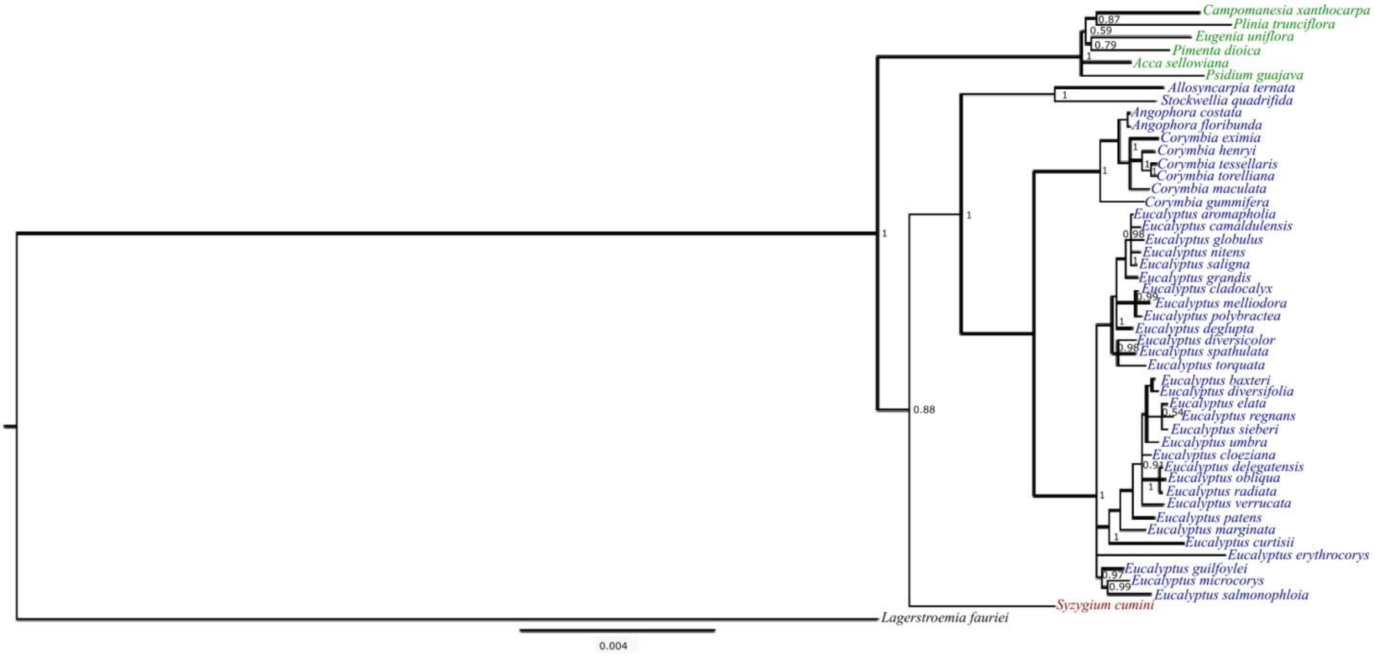


**Figure S2** - Bayesian phylogeny based on six most variable genes (*ycf2*, *atpA*, *rpoC2*, *pcbE*, *ndhH* and *rps16*) on chloroplast sequences of 48 Myrtaceae species and the outgroup *Lagerstroemia fauriei* (Myrtales: Lythraceae; KT358807). Branch length is proportional to the inferred divergence level. The scale bar indicates the number of inferred nucleic acid substitutions per site. Species in green are from tribe Myrteae, in blue are from tribe Eucalypteae, in brown from tribe Syzygieae.
