# Supplementary material for: Molecular relationships of Campomanesia xanthocarpa within Myrtaceae based on the complete plastome sequence and on the plastid ycf2 gene
Source: Genet Mol Biol. 2020 Jun 10;43(2):e20180377. doi: 10.1590/1678-4685-GMB-2018-0377 (PMC7288672; doi:10.1590/1678-4685-GMB-2018-0377)
Supplement: Supplementary file 4 [file 1415-4757-GMB-43-2-e20180377-suppl3.pdf]

Genetics and Molecular Biology 43, 2 (2020)

Supplementary Material to "Molecular relationships of *Campomanesia xanthocarpa* within Myrtaceae based on the complete plastome sequence and on the plastid *ycf2* gene"

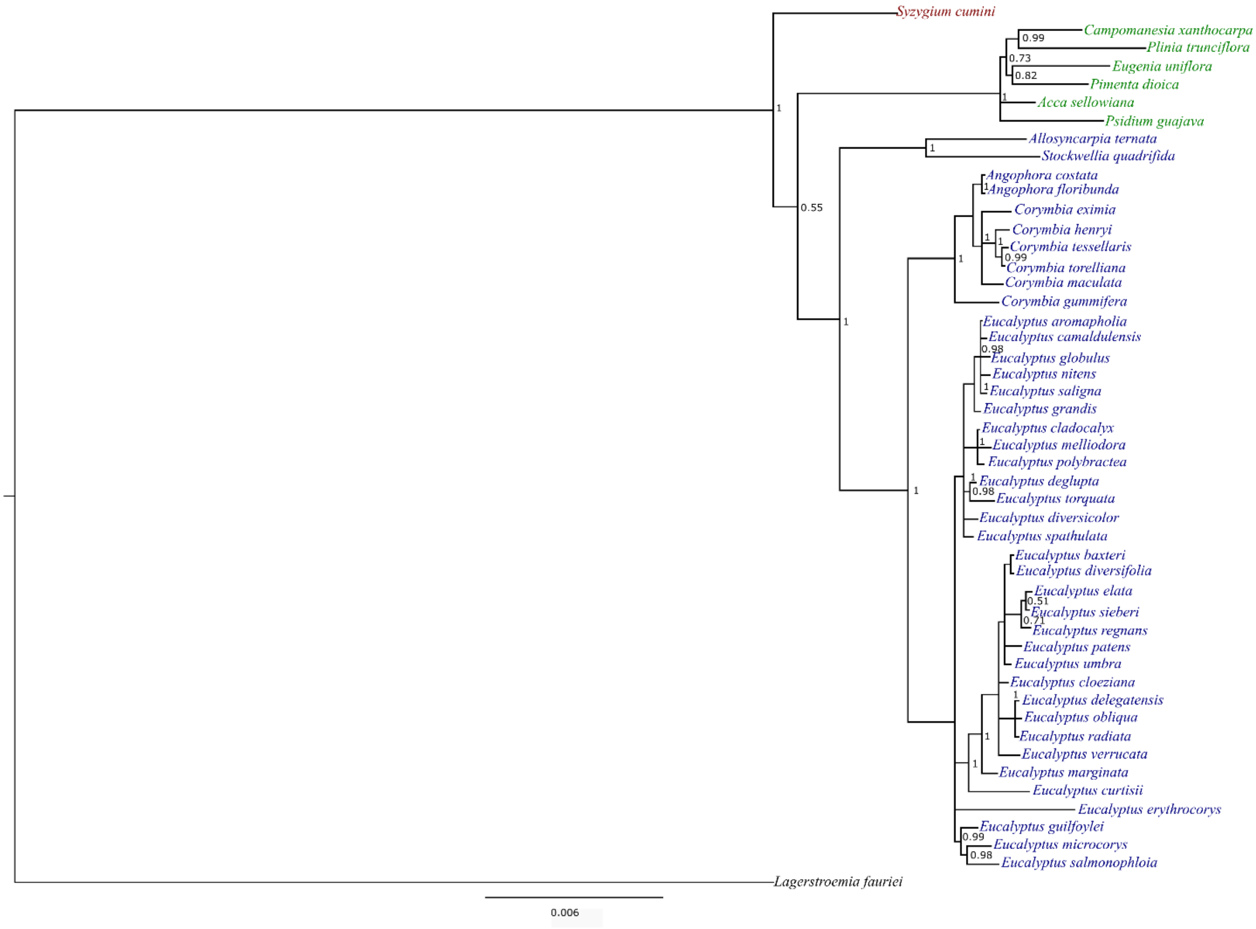

Figure S3 - Bayesian phylogeny based on five most variable genes (*atpA*, *rpoC2*, *pcbE*, *ndhH* and *rps16*) on chloroplast sequences of 48 Myrtaceae species and the outgroup *Lagerstroemia fauriei* (Myrtales: Lythraceae; KT358807). Branch length is proportional to the inferred divergence level.
